# Conservative treatment of lymphedema: the state of the art

**DOI:** 10.1590/1677-5449.200091

**Published:** 2021-10-11

**Authors:** Anke Bergmann, Jaqueline Munaretto Timm Baiocchi, Mauro Figueiredo Carvalho de Andrade

**Affiliations:** 1 Instituto Nacional de Câncer, Rio de Janeiro, RJ, Brasil.; 2 Instituto Oncofisio, São Paulo, SP, Brasil.; 3 Universidade de São Paulo – USP, Faculdade de Medicina, Departamento de Cirurgia, São Paulo, SP, Brasil.

**Keywords:** lymphedema, combined modality therapy, physical therapy specialty, rehabilitation, treatment, linfedema, terapia combinada, fisioterapia, reabilitação, tratamento

## Abstract

This article aims to discuss the possibilities of conservative and non-pharmacological treatments for lymphedema. A non-systematic review of the literature was carried out, including studies involving human subjects with different types of lymphedema. Several approaches to lymphedema treatment have been reported and Complex Decongestive Therapy (CDT) has been considered the most effective treatment for limb lymphedema. Other conservative treatments have been proposed such as Taping, Extracorporeal Shock Wave Therapy, Acupuncture, Photobiomodulation Therapy, Endermologie, Intermittent Pneumatic Compression, and Low-frequency, Low-intensity Electrotherapy. The choice of the therapeutic approach to be employed should consider lymphedema characteristics, the therapist's experience, and the patient's wishes. In addition, since this is a chronic condition, the patient must adhere to the treatment. To this end, the therapeutic proposal may be the key to better control of limb volume.

## Resumo

Este artigo tem como objetivo discutir as possibilidades de tratamentos conservadores e não medicamentosos para o linfedema. Foi realizada uma revisão não sistemática da literatura, com a inclusão de estudos realizados em humanos com diferentes tipos de linfedema. Várias abordagens de tratamento foram relatadas, sendo a terapia descongestiva complexa considerada o tratamento mais eficaz para o linfedema de membros. Outros tratamentos conservadores foram propostos, como *taping*, terapia por ondas de choque extracorpórea, acupuntura, fotobiomodulação, endermologia, compressão pneumática intermitente e eletroterapia de baixa intensidade e baixa frequência. A escolha da abordagem terapêutica a ser empregada deve considerar as características do linfedema, a experiência do terapeuta e os desejos do paciente. Além disso, como é uma condição crônica, o paciente deve aderir ao tratamento. Para isso, a proposta terapêutica pode ser a chave para um melhor controle do volume do membro.

## INTRODUCTION

Several approaches to lymphedema treatment have been reported in the literature. Among conservative treatments, complex decongestive therapy (CDT) (also known as complex decongestive physiotherapy, combined physical therapy, and complex physical therapy, among others) is backed by longstanding experience and is noteworthy as the best approach for reducing upper limb lymphedema volume after breast cancer and lower limb lymphedema volume after gynecological cancer, as well as in lymphedema of other etiologies.^1^^-^ 3

Other conservative approaches have been proposed for treatment of lymphedema, mainly as adjuvants to CDT, including intermittent pneumatic compression (IPC),^4^ taping,^5^ extracorporeal shock wave therapy,^6^ photobiomodulation therapy,^7^ and acupuncture,^8^ among others.

In this paper, we will discuss conservative and non-pharmacological treatments that can be used to reduce the volume of limbs with lymphedema and maintain the reductions.

## COMPLEX DECONGESTIVE THERAPY (CDT)

CDT consists of two treatment phases and four components, namely skin care, manual lymphatic drainage (MLD), compression therapy, and exercises. The first phase of this treatment aims at the maximum reduction of limb volume, with skin care, MLD, multilayer wrapping, and exercises performed in daily sessions lasting from four to six weeks. The maintenance phase (second phase) begins immediately after this phase. Its objective is to conserve and optimize the results obtained in the initial phase and it consists of fitting of elastic garments, exercises, skin care, and MLD when necessary.^9^

Studies carried out with different lymphedema etiologies have shown that CDT reduces limb volume and symptoms and improves quality of life and patients report satisfaction with the treatment received, so this therapy is currently considered the gold standard treatment.^10^^-^ 13

In women with breast cancer-related lymphedema (BCRL), the response to CDT treatment is associated with weight control, lymphedema grade, physical activity, and adherence to the use of compression therapy.^14^^,^ 15 Quality of life and social support were not predictors of better therapeutic response in this population.^16^^,^ 17

### Manual Lymph Drainage

**Figure 1:**
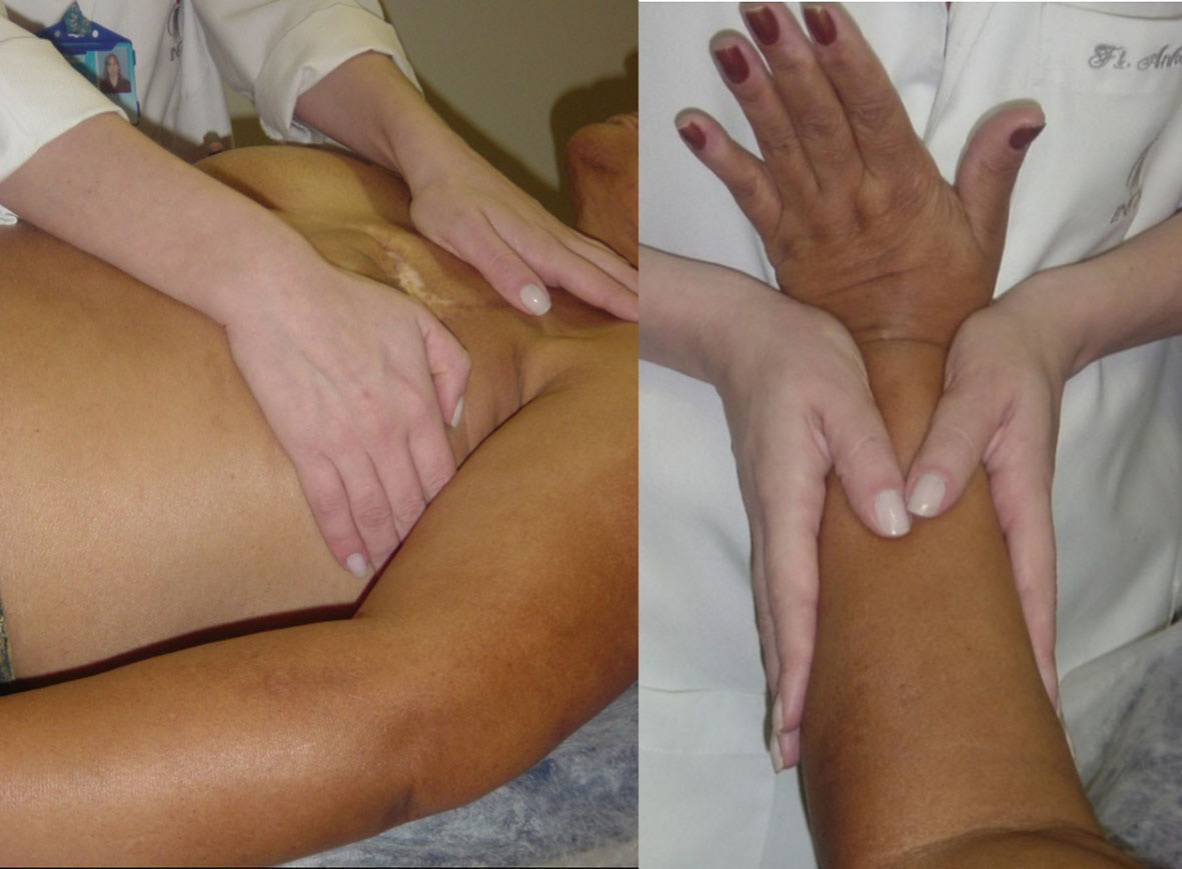
Manual lymph drainage in a patient with breast cancer-related lymphedema.

MLD consists of a specific manual therapy performed on the superficial lymphatic system, by means of precise, light, smooth, slow, and rhythmic maneuvers, which obey lymphatic system anatomy and physiology (Figure 1).

Its main objectives are to increase absorption of liquid and proteins from the interstitium by the lymphatic capillaries, increase the contractility of the lymphatic collectors, and increase liquid lymph node absorption, thus increasing the amount of liquid that returns to the venous system through the lymphatic system.^18^ In addition, because they are maneuvers that involve superficial touching, MLD can also promote quality of life improvement, sleep improvement, and reduction of pain, anxiety, and other symptoms.^19^^-^ 21

However, the effectiveness of manual lymphatic drainage for reducing lymphedema is not yet clear in the scientific literature.^19^ A clinical trial was conducted in Brazilian women with BCRL who underwent CDT and were randomized into two groups: with or without MLD. Both groups displayed reduction in limb volume at the end of the first treatment phase, with no difference between them.^22^ Other randomized clinical trials have reported similar results, with no difference in response to CDT with or without MLD.^23^^-^ 24

Studies have shown that MLD is a safe treatment and, when performed, can offer additional benefits to CDT, promoting better maintenance of the effects of compressive therapy, better quality of life, and improvement of symptoms resulting from lymphedema and areas of lymphostatic fibrosis.^11^^,^ 25 ^-^ 27

### Compression therapy

**Figure 2:**
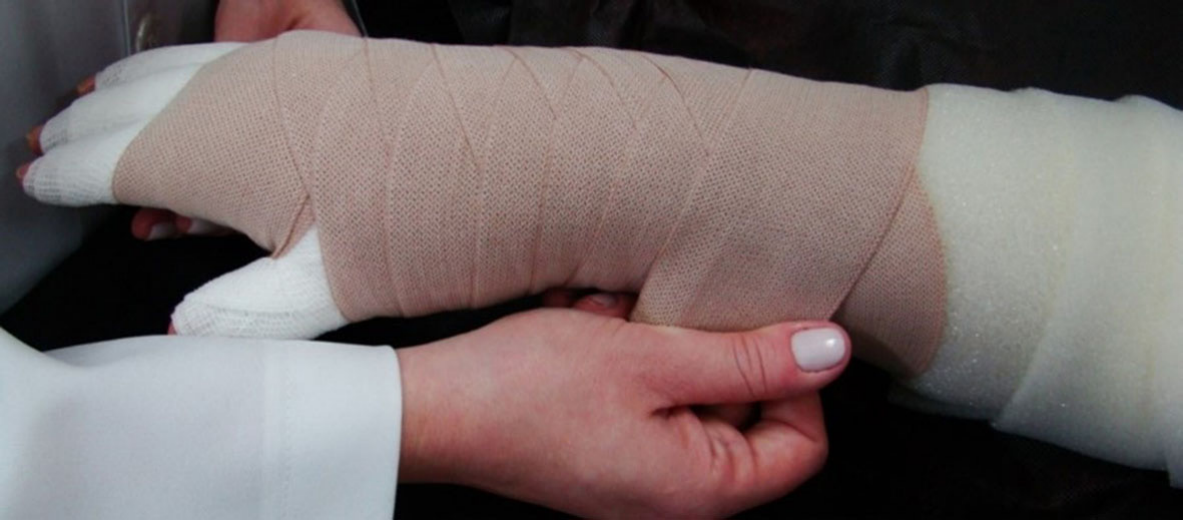
Multi-layer bandaging in a patient with breast cancer-related lymphedema.

Compression therapy is performed using multi-layer bandaging (Figure 2), adjustable compression devices, and elastic garments. It is considered the main resource for lymphedema treatment, both in the volume reduction phase and in the maintenance phase.^9^

The effects of compression therapy on the lymphatic system include reduction of excess interstitial fluid due to decreased blood ultrafiltration, greater resorption, and improved muscle pumping.^28^ In the venous system, compression therapy reduces reflux and improves venous return, decreases venous hypertension, improves the calf muscle pump, and can improve the clinical conditions of venous ulcers. In addition to these effects, compression therapy also acts on trophic changes, by releasing anti-inflammatory mediators, minimizing areas of interstitial fibrosis.^28^ Compression therapy can have favorable results, improving pain, functionality, and quality of life.^28^

When the therapeutic objective is to reduce limb volume, the multi-layer bandage treatment leads to the best clinical response. The pressure exerted on the limb during muscle contraction (working pressure) will depend on the type of material, the degree of extensibility or stretching (tension applied during the bandaging), the force exerted by the bandage (the number of layers), and the conditions of the material (usage time, washing method). For lymphedema treatment, use of short extensibility bandages is recommended because they produce greater working pressure. The greater the tissue pressure in the interstitium imposed by compression, the better the absorption of interstitial fluid.^29^ However, continuous high pressure can lead to blood capillary occlusion, resulting in pain and skin damage.^30^ Determination of the ideal pressure must take into account lymphedema type and severity, presence of any fibrosis, and skin conditions.^31^

In the second lymphedema treatment phase, wearing of elastic garments is indicated. For each clinical situation, it is necessary to assess the appropriate compression class, which depends on the physical and dynamic aspects of the fabrics (elasticity and stiffness) and also on the specific characteristics of each patient (skin texture, limb size, edema location, presence of lymphostatic fibrosis, and functionality of the affected limb).^28^

Another option for compression therapy that can be used both in the reduction of limb volume phase and in the maintenance phase is adjustable compression devices. These consist of a garment made of low elasticity fabric that wraps around the limb with lymphedema, attached with adjustable VELCRO®. These self-adjusting devices allow patients to maintain great compression as the limb volume decreases.^32^ Although VELCRO® devices are not better than bandage wrapping, they may be an alternative option for patients who do not adhere to other forms of compression, those with significant wounds or skin changes, or for financial reasons.^33^^-^ 37

### Exercises

Active exercises are indicated for patients with lymphedema in order to increase venous return and lymphatic absorption through muscle pumping. These results are better when performed with some form of external compression.^9^^,^ 38 ^,^ 39

Several different types of exercises have been deemed safe in patients with lymphedema, including water exercises, stretching, Pilates, swimming, walking, resistance exercises, yoga, weight training, and aerobic exercises.^40^^,^ 41 A study that evaluated the performance of passive exercises in combination with CDT showed no difference in the outcomes analyzed.^42^

The choice of exercise should take into account the patient's preference. Whenever possible, patients should be instructed to perform activities of daily living as a form of exercise, prioritizing activities with greater energy expenditure.

### Skin care

Patients with lymphatic insufficiency may present skin changes such as thickening, hyperkeratosis, papillomatosis, skin fold deepening, skin fissures, dermal fibrosis, and lymphorrhea, among others. These complications are associated with higher risk of infection and worsening of lymphedema grade, functionality, and quality of life.^43^^,^ 44

Therefore, skin care is essential in the treatment of lymphedema and must be performed during all phases of CDT. Patients should be instructed to perform daily hygiene measures with careful washing and daily moisturizing and to avoid skin damage or trauma.^44^^,^ 45

## OTHER COMPLEMENTARY CONSERVATIVE TREATMENTS

### Taping

**Figure 3:**
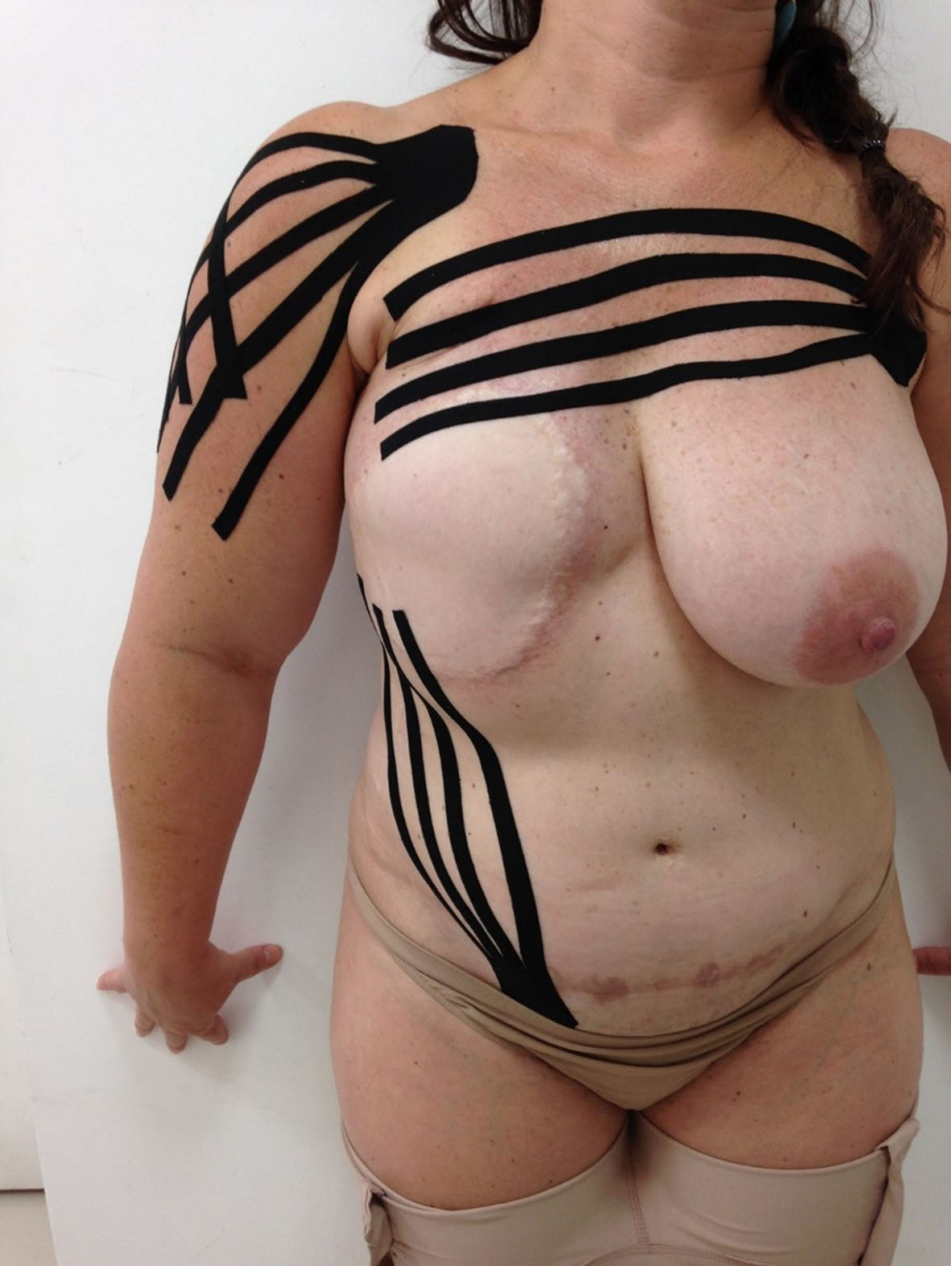
Taping in a patient with breast reconstruction and lymphedema.

Two meta-analyses have been published recently analyzing taping for lymphedema treatment after breast cancer, but without standardization of the form of application.^46^^,^ 47 The most usual application is along the lymphatic system path (Figure 3), in order to facilitate interstitial fluid reabsorption through creation of space, caused by skin stretching during muscle contraction.^46^

Meta-analyses of clinical trials showed that taping is not more effective for reducing limb volume than bandaging, although better quality of life, comfort, and convenience were observed in the group to whom taping was applied. Studies conclude that taping may be an alternative way of treating lymphedema for patients who have some contraindication to compressive therapy.^5^^,^ 47 Despite being considered a safe technique, patients can suffer skin lesions caused by application of taping.^48^

### Extracorporeal Shock Wave Therapy (ESWT)

Extracorporeal shock wave therapy (ESWT) is a type of mechanical energy that penetrates the tissue, causing a cavitation phenomenon, leading to microcracks in the inflamed tissue which, in turn, releases local anti-inflammatory substances and stimulates local microcirculation.^6^

In an experimental study using a rabbit ear model of secondary lymphedema, it was observed that extracorporeal shock wave therapy enhanced expression of vascular endothelial growth factor-C and of its receptor, VEGF receptor 3, and increased formation of lymphatic vessels.^49^ A similar result was observed in a rat tail model of lymphoedema, in which lymphangiogenesis induction and lymphedema improvement occurred.^50^

Two pilot studies that applied extracorporeal shock wave therapy in women with BCRL reported reduced limb volume and absence of adverse events.^51^^,^ 52 An improvement in the subcutaneous and dermis layer was observed after ESWT application in two cases assessed with computed tomography.^53^

Extracorporeal shock wave therapy may add some benefit, but due to the absence of randomized clinical trials, there is not enough evidence to claim that ESWT should be incorporated into lymphedema treatment.

### Acupuncture

Two meta-analyses including women with BCRL demonstrated better objective and subjective outcomes in groups undergoing CDT with acupuncture when compared to groups with CDT without acupuncture or other treatment modalities.^8^^,^ 54

A case report describing 12 women with lymphedema after gynecological cancer who underwent acupuncture and moxibustion immediately after lymphedema occurrence reported total improvement in symptoms in seven cases of mild lymphedema, marked improvement in six cases and worsening of lymphedema in one case.^55^

### Photobiomodulation therapy

**Figure 4:**
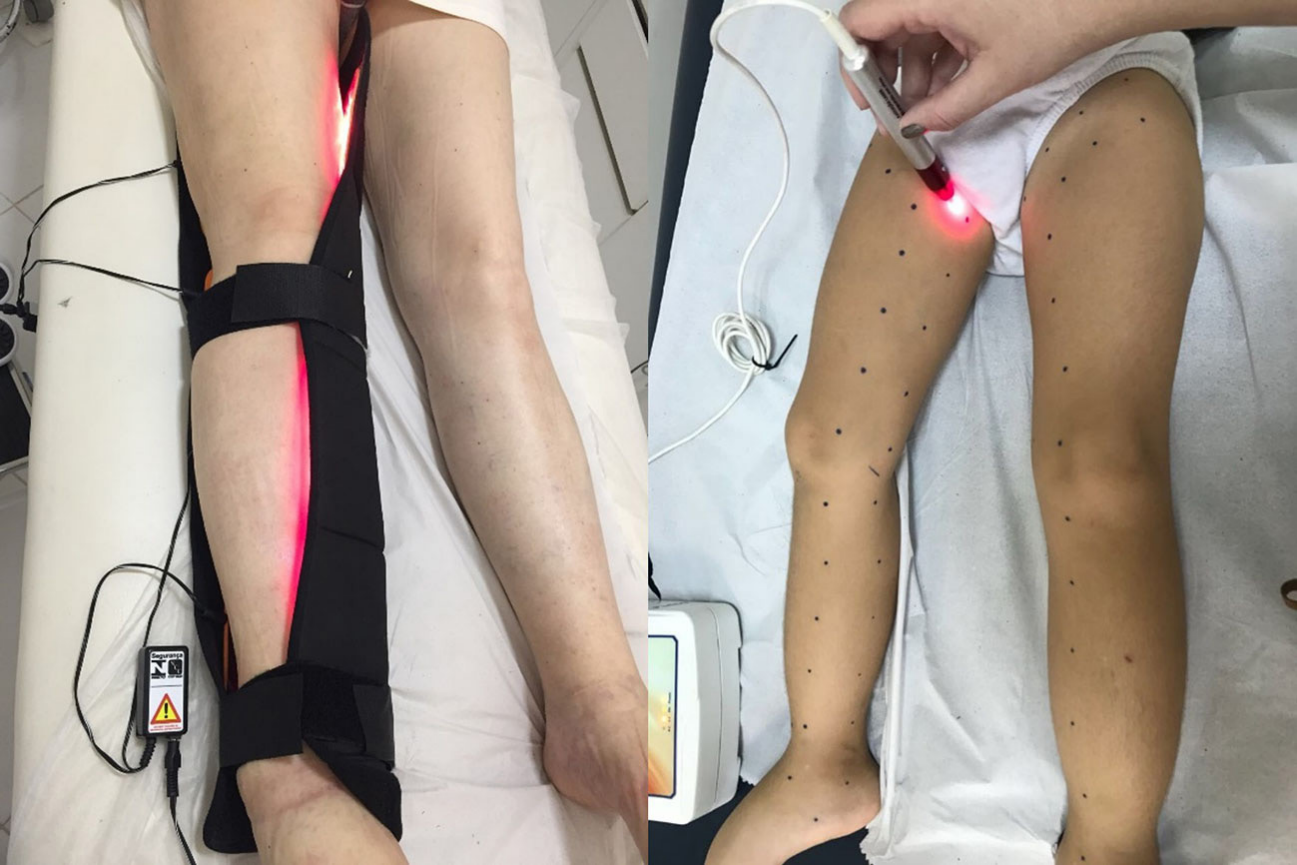
Photobiomodulation therapy in a patient with lower limb lymphedema.

Over the past two decades, photobiomodulation (PBM), which was formerly known as low level laser therapy (LLLT), has been researched for management of lymphedema. PBM is a non-invasive form of phototherapy that utilizes wavelengths of light between 650 and 1000 nm, ranging from red light to near infrared light (NIR), to deliver low-level irradiance doses to the target tissue (Figure 4). It has been used to reduce inflammation, promote lymph vessel regeneration, improve lymphatic motility, and treat and prevent tissue fibrosis.^56^

Systematic reviews^7^^,^ 57 ^-^ 59 studied the effects of PBM for BCRL, providing strong evidence in its favor (PBM over sham in terms of reduction in limb edema at short-term follow-up). The infrared wavelengths (808-905 nm) have been most commonly employed to date, and the energy densities reported that had positive outcomes were in the range of 1.5 J/cm2 to 2.4 J/cm2.

A previous systematic review evaluating a series of conservative therapies has demonstrated that PBM yielded a similar percentage of volume reductions (approximately 11%) compared to compression garments or bandages.^27^ However, some studies found conflicting evidence regarding the effectiveness of PBM compared to the gold standard treatment, CDT, for reducing limb circumference and pain intensity.^60^^-^ 62

Several studies using low-power sources and wavelengths have been published reporting the safety of red and NIR laser irradiation in terms of stimulation of tumor cell growth.^63^^-^ 69

Cialdai et al,^70^ showed that irradiation with a high-power dual wavelength (808 nm and 905 nm) NIR laser did not affect the behavior of human dermal fibroblasts and breast adenocarcinoma cell lines in terms of proliferation, cell cycle progression, apoptosis, or cloning efficiency. These results are consistent with the possibility of safely administering NIR laser irradiation for management of secondary lymphedema due to cancer.^68^

### Endermotherapy – vacuum suction therapy

Vacuum massage is also known as depressomassage, vacuotherapy, endermotherapy, or Endermologie®. It is a non-invasive mechanical massage technique performed with a mechanical device that lifts the skin by means of suction, creates a skin fold, and mobilizes it.^71^

Some studies of its use with burn scars and lipodistrophy showed that vacuum massage may release the mechanical tension associated with scar retraction and thus induce apoptosis of myofibroblasts. Improvements in tissue hardness and skin elasticity were the two most observed effects.^72^

Studies using endermotherapy have shown that it improves superficial lymphatic drainage and lymphatic transport capacity, decreases fibrotic induration, heaviness, tightness, and functional discomfort.^73^^,^ 74 Malloizel-Delaunay J et al.^75^ are conducting a phase II RCT of endermotherapy for BCRL lymphedema, but no data has been published yet.

### Intermittent Pneumatic Compression (IPC)

**Figure 5:**
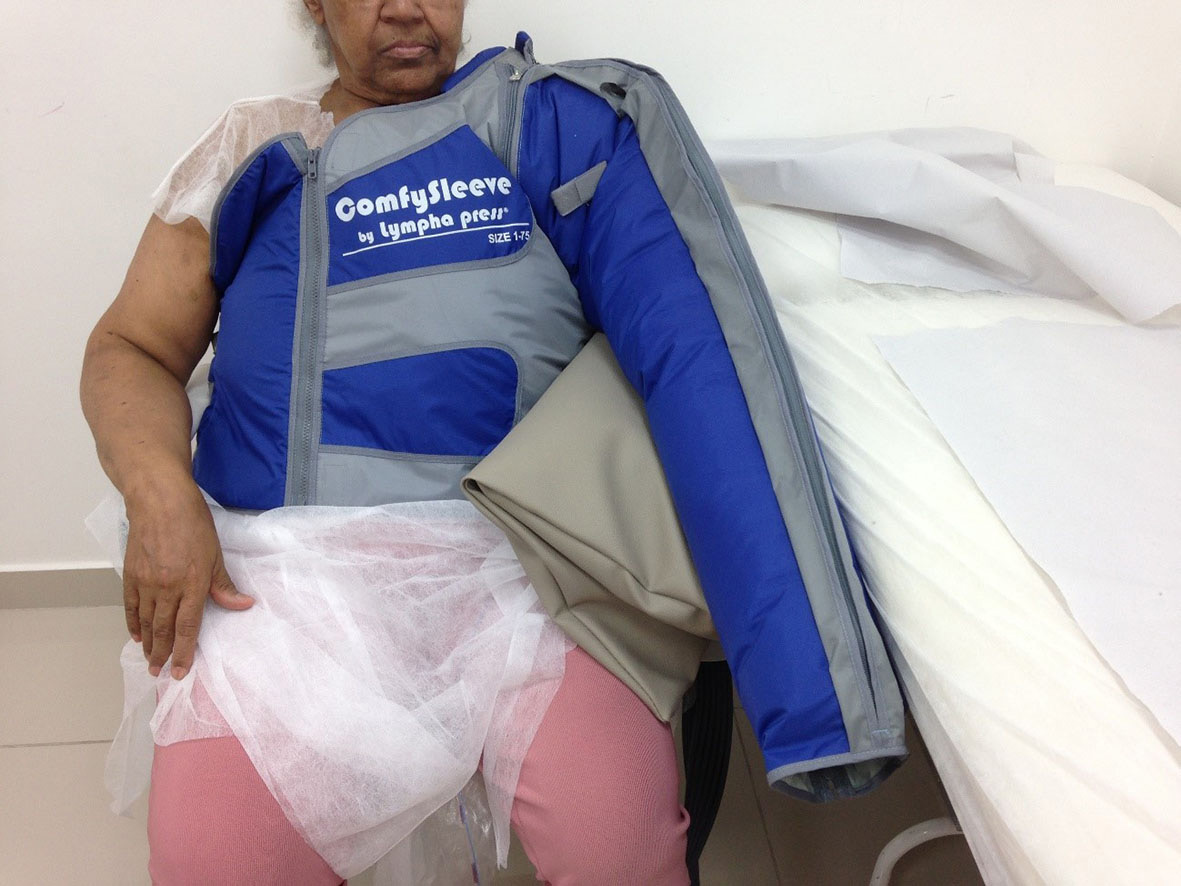
Intermittent pneumatic compression in a patient with breast cancer-related lymphedema.

Intermittent pneumatic compression pump (IPC) devices are pneumatic cuffs connected to pumps that mimic the naturally-occurring pumping effect of muscles contracting around peripheral lymphatics (Figure 5). They were developed to replicate a therapist’s hands when performing manual techniques, utilizing low pressure with short repetitive applications, moving progressively along a limb to simulate manual lymph drainage (MLD), with garments that extend to the root of the limb, to clear the pathway for drainage.^76^

A variety of pumps are available. Pneumatic compression device product classifications include nonsegmental and segmental and home or professional models, for half or full limb, with or without calibrated gradient pressure. Devices differ with respect to the number of chambers, time of inflation, deflation, regulation of inflation pressure, and calibrated gradient pressure, as well as in terms of garment shapes.^77^

Regarding the parameter settings on IPC machines, older studies used higher pressures (100–150 mmHg), while nearly all studies from recent years have applied pressures ranging from 30 to 60mmHg. There is limited low-to-moderate quality evidence for application of 45–60 minutes of 30–60mmHg using multicell, sequential IPC programs for management of upper and lower limb lymphedema.^78^^,^ 79

A previous systematic review indicated that IPC devices are well-tolerated in low-to-moderate pressure ranges. Moreover, devices enable application of compression in the patient’s home. IPC is also a safe and effective intervention and may constitute an acceptable home-based treatment modality in addition to wearing compression garments.^77^ For management of BCRL, IPC resulted in significant alleviation of edema and subjective symptoms, but addition of IPC failed to show superiority compared to CDT alone.^80^

A series of clinical trials and systematic reviews have tried to investigate the benefits of IPC. However, the results have been controversial and no final conclusion has been reached on the influence of IPC on lymphedema.^81^^-^ 83 More recently, Zaleska et al.^84^ reported that IPC devices can lower tissue fluid pressures, increase flow volume, and decrease skin stiffness, moving subcutaneous extracellular water away to proximal regions of the limb. There is limited low to moderate quality evidence for use of IPC in lymphedema treatment, but home use should be considered for the maintenance phase.

### Low-frequency, low-intensity electrotherapy

Low-frequency, low-intensity electrotherapy (Deep Oscillation Therapy®) is an adjuvant vibrational technology that consists of applying an intermittent electrostatic field with low intensity (U = 100–400V; I = 150μA) and extremely low frequency (30–200Hz, rectangular, biphase) to the target area. The field electrostatically attracts and releases the patient’s tissues at the selected frequency, resulting in deep and lasting resonance vibration. Both the patient and the therapist are connected to the device, which serves as a source of tension with high internal resistance. The treatment technique includes use of a pair of maniples and electrodes which are applied to the patient’s skin in areas corresponding to lymph node stations.^85^^-^ 87

The field is applied using a slow, circular motion, without pressing, following lymphatic routes. It provokes cyclic movement in deep tissues, leading to mechanical pumping and redistribution of fluids. Patients may feel a slight tingling or a slight sensation of heat during treatment, but it is absolutely painless. It is claimed that the effects of manual lymph drainage are intensified by deep oscillations.^88^

Deep oscillation massage is used to stimulate absorption of edema, to reduce pain, and to alleviate wound healing, due to its anti-inflammatory and antifibrous effects.^85^^-^ 87 ^,^ 89 ^,^ 90

Ohkuma^91^ presented the first published data on the effect of electric fields in 10 lower limb lymphoedema patients. He reported decrease in leg volume and no adverse sequelae of treatment, except for one patient who developed transient skin erythema. Ricci^92^ reported volume reduction in 57% of their 28 upper-limb secondary lymphoedema patients treated with low-frequency, low-intensity electrotherapy, but there is no information about the amount of change. Jahr et al.^93^ presented use of this technique to stimulate lymphatic flow by deep resonance vibration. Their study reported alleviation of pain and reduction of swelling in patients with BCRL. Belmonte et al.^94^ compared Deep Oscillation®️ to MLD and observed that the results were the same, since there were no statistical differences between groups.

In conclusion, deep oscillation therapy produces a tissue-relaxing, moderate vasoconstriction effect, local edema reabsorption, and fibrosis reduction. Information is not available on the safety or tolerability of this technique.^85^^-^ 94

## CONCLUSION

Lymphedema is a chronic condition and since no method to restore normal lymphatic function is available, lifelong control is therefore mandatory. Complex decongestive therapy is considered the treatment of choice for most lymphedema patients, but it is dependent on therapist skill, patient compliance, and availability of equipped facilities. Nevertheless, acquisition of robust data to recommend its universal use is hindered by treatment characteristics themselves. A large series of alternative conservative approaches have been proposed, but consistent data demonstrating their safety, consistency of results, and long-lasting control of the lymphedema patient are still lacking. Unfortunately, unlike many disorders, lymphedema patients constitute an array of varied presentations, displaying wide variability of demographic and etiological characteristics, degree of tissue changes, and severity of diseases, thus challenging researchers attempting to perform well-conducted randomized controlled clinical trials.
